# Recent Advances in Flexible Multifunctional Sensors

**DOI:** 10.3390/mi14112116

**Published:** 2023-11-18

**Authors:** Ya Chang, Xiangyu Qi, Linglu Wang, Chuanbo Li, Yang Wang

**Affiliations:** 1 School of Science, Minzu University of China, Beijing 100081, China; 2 Optoelectronics Research Centre, Minzu University of China, Beijing 100081, China

**Keywords:** multifunctional sensor, sensing materials, wireless communication, electronic skin

## Abstract

Wearable electronics have received extensive attention in human–machine interactions, robotics, and health monitoring. The use of multifunctional sensors that are capable of measuring a variety of mechanical or environmental stimuli can provide new functionalities for wearable electronics. Advancements in material science and system integration technologies have contributed to the development of high-performance flexible multifunctional sensors. This review presents the main approaches, based on functional materials and device structures, to improve sensing parameters, including linearity, detection range, and sensitivity to various stimuli. The details of electrical, biocompatible, and mechanical properties of self-powered sensors and wearable wireless systems are systematically elaborated. Finally, the current challenges and future developmental directions are discussed to offer a guide to fabricate advanced multifunctional sensors.

## 1. Introduction

**Figure 1:**
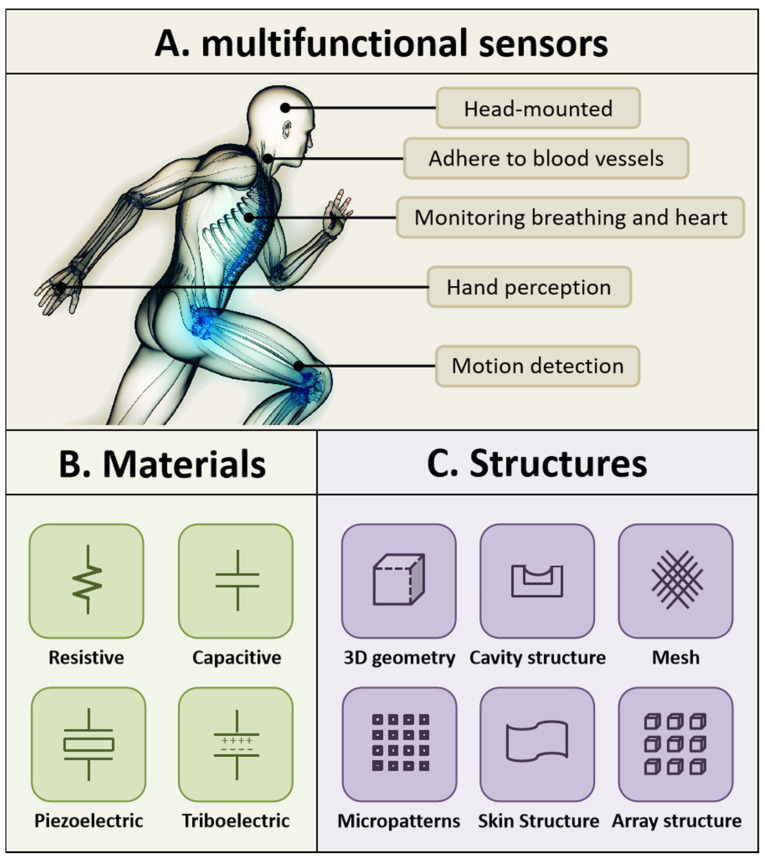
Multifunctional sensor. (**A**) Some common applications of multifunctional sensors. (**B**) Common materials used for multifunctional sensors. (**C**) Common structures of multifunctional sensors.

**Figure 2:**
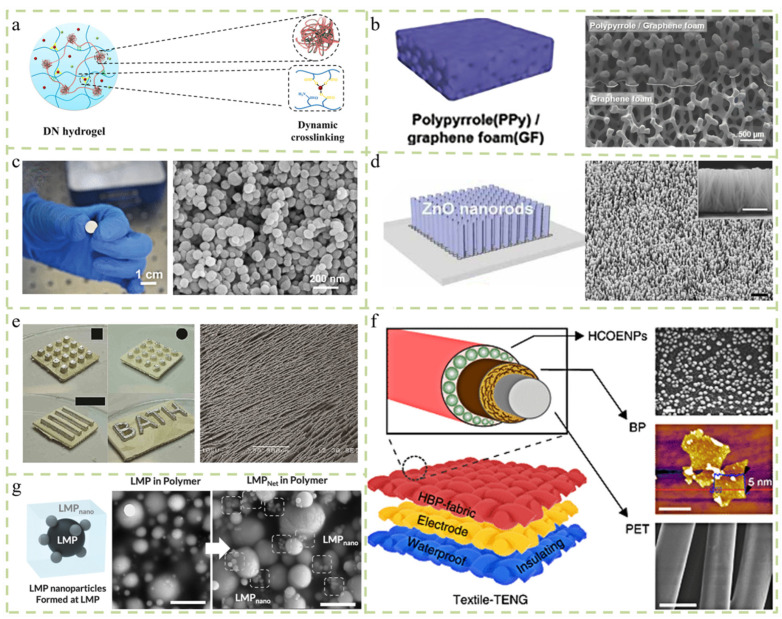
Types of sensor materials. (**a**) Freeze-resistant, highly sensitive strain and pressure sensors assembled with dynamically cross-linked, ionically conductive hydrogels [6]. (**b**) Based on the properties of graphene foams, multifunctional sensors are manufactured for wireless power transfer wearable devices [10]. (**c**) Development of a thermopiezoelectric sensor system for energy conversion based on ferroelectric materials [13]. (**d**) Application of ZnO nanorods in energy harvesting and tactile sensing [15]. (**e**) Self-powered flexible and highly active pressure and shear sensors based on frozen cast ceramic polymer structures [18]. (**f**) Washable skin-driven textile-based triboelectric nanogenerators with black scales as the main material [23]. (**g**) Friction electric nanogenerator of textiles driven by skin contact movement [25].

Wearable electronics have motivated comprehensive research on flexible sensors that can respond to stimuli, such as stretching, pressure, and temperature. Flexible sensors convert stimuli into electrical signals while worn, and they can potentially be used in robotic hands, electronic skin, and medical diagnoses (Figure 1A) [1]. However, most of these sensors detect single stimuli and are thus unable to meet specific demands that require perceiving multiple stimuli at the same time. Therefore, over the past decade, multifunctional flexible sensors that can perceive two or more external stimuli in a single unit have attracted substantial attention owing to their suitability for expansive applications. Multifunctional sensors usually include the following: (1) functional materials to respond to different stimuli (Figure 1B); (2) advanced structures to recombine sensing materials (Figure 1C); and (3) intelligent systems to decouple overlapped signals. Several novel materials, from ultrathin silicon to ionic liquid, have been used to fabricate multifunctional sensing elements. For example, Park et al. developed multimodal receptors with a simple electrode–electrolyte–electrode structure that can simultaneously detect temperature and strain [2]. Moreover, there have been many studies that have developed excellent designs, including micro-patterned structures [3], porous layers, and combined approaches to improve the performances of sensors [4].

Recently, several reviews have extensively presented the progress of flexible pressure or strain sensors [5]. Therefore, this review investigates the recent strategies that have been developed in relation to multifunctional flexible sensors for various applications. First, we summarize the materials that are typically used for multimodal sensors. We also analyze different structures, transduction mechanisms, and signal recording methods to improve sensitivity, linearities, and detection ranges. This review then discusses specific self-powered and wireless sensors, as well as their applications. Finally, we discuss the current challenges in the field of multifunctional sensors and envision future development trends for new flexible sensors. 

## 2. Materials

The functional materials in multifunctional sensors directly affect their sensing parameters, such as linearity, detection range, and sensitivity. In addition, these materials should be safe, comfortable, and reliable. In the following section, various advanced materials are presented along with their electrical properties, with explicit consideration given to their functionality in wearable electronics.

### 2.1. Hydrogel Material

Hydrogel material is a kind of elastomer that integrates hydrogen bonds, electrostatic interaction, and hydrophobic effects into the polymer network. Conductive particles can be added to adjust the conductivity of the hydrogel. The conductive hydrogel can detect changes in response to mechanical deformation or pressure by converting external mechanical stimuli into electrical signals. Liu et al. used an ion-conductive chitosan–poly double-network hydrogel to fabricate a strain and pressure sensor with double dynamic cross-linking characteristics, freezing resistance, high sensitivity (sensitivity = 0.208 KPa^−1^), good tensile strength (ε ≈ 450%), supercompression (ε = 98%), rapid self-healing ability, and significant fatigue resistance (1000 cycles) (Figure 2a) [6]. Table 1 summarizes the material properties. In practical applications, the development of conductive hydrogels with dynamic cross-linking can solve the problems of plastic deformation and structural damage under cycles and high deformation or high pressure. 

In addition, low temperatures can lead to the formation of ice crystals in the hydrogel, resulting in a reduction in sensing sensitivity. The introduction of polyelectrolyte and glycerol into the hydrogel can enable it to show excellent elongation, conductivity, and self-healing properties even at −20 °C [7]. Xu et al. created an antifreeze hydrogel sensor by introducing antifreeze proteins into hydrogels inspired by fish [8].

### 2.2. Graphene Foam

A new kind of carbon material, graphene, has the advantages of good electrical conductivity and high chemical stability [9]. However, graphene has some drawbacks, such as the difficulty and expense involved in producing graphene on a large scale. Graphene has high reactivity with oxygen and heat. Although graphene has good electrical conductivity, it is not particularly stable. Graphene oxide will destroy the performance of graphene itself, resulting in the loss of electrical conductivity. Currently, large amounts of graphene can be prepared using the CVD method. However, in an aerobic environment, its stability still needs to be tested. The conversion of two-dimensional (2D) graphene into graphene foam (GF) is a subject of current mainstream research. As a new type of carbon-based chemical material, GF has attracted much attention because of its highly microporous structure, excellent electrical conductivity, and large surface area. Its 3D porous structure is also suitable for use as a scaffold material for monolithic composite electrodes. However, graphene foam material is not perfect. Because of its brittleness, the ease with which it collapses during processing, and the potential for its 3D structure to be destroyed, its practical applications are greatly limited. The need to improve the mechanical properties of graphene has led to increasing interest in the potential properties of graphene foam, which is also a technical problem to be solved at present. Park et al. developed a dual-mode sensor, of which a polydimethylsiloxane-coated microporous polypyrrole and graphene foam composite are the basic materials. The sensor can detect pressure and temperature according to current and voltage changes, and the functions do not interfere with each other [10]. The manufactured dual-mode sensor provides high sensitivity (2.01 KPa^−1^), a fast response time (8.3 s), swift recovery, and high durability during 10,000 pressure loading cycles at 1 KPa of pressure (Figure 2b).

### 2.3. Ferroelectric Materials

Ferroelectric materials with spontaneous polarization are widely used in communication systems, microelectronics, and integrated optics due to their ferroelectric, dielectric, piezoelectric, pyroelectric, and electro-optical properties [11]. Barium titanate (BaTiO_3_) is widely used in electronic ceramics. It has high dielectric ceramics, low dielectric loss, and excellent pyroelectric and piezoelectric properties, and it exhibits significant sensitivity to temperature and strain [12]. Song et al. developed a novel array sensor system to sense finger-induced temperature and pressure changes in real time using BaTiO_3_ ferroelectric materials [13]. The voltage signal of this device increases with the increase in the temperature difference, with a sensitivity of approximately 0.048 V °C^−1^. As the applied pressure increases, the sensitivity is approximately 0.044 V KPa^−1^. The system has potential applications in machine intelligence and human–computer interaction (Figure 2c). There are many more typical ferroelectric materials, such as potassium dihydrogen phosphate, ferroelectric thin films, etc. Wenru Li et al. reported on a self-powered flexible mechanical sensor prepared by depositing molecular ferroelectrics on a porous polyurethane matrix. It can detect a pressure of 3 Pa and a strain of 1%, and it has high cyclic stability. It also has potential applications in future interactive electronic and robotic systems [14].

### 2.4. Nano Zinc Oxide

As a functional material with both semiconductor and piezoelectric properties, nano zinc oxide (ZnO) material has good thermal stability and biocompatibility and it is widely used in the preparation of various functional devices (Figure 2d). Zinc oxide has important research value in various fields, such as optoelectronics, piezoelectricity, pyroelectricity, and ferroelectricity, because of its diverse preparation functions, excellent performance, low price, non-toxicity, and various preparation methods. The concept of nanogenerators was first successfully developed on ZnO nanorods, realizing the conversion of mechanical energy into electrical energy [15].

### 2.5. Ceramic–Polymer Composites

Crystals with a piezoelectric effect have low symmetry and will deform under the action of external force. At this time, the relative displacement of positive and negative ions in the unit cell appears at the centers of the positive and negative charges with the macroscopic electric polarization of the crystal. The surface density of the charge on the crystal surface is equal to the projection of polarization on the surface normal. When the piezoelectric material is deformed under pressure, different charges will appear on both ends. Conversely, when a piezoelectric material is polarized in an electric field, the material will deform due to the displacement of the charge center [16]. Electroceramic materials have high dielectric properties, piezoelectricity, and electromechanical coupling. These characteristics can adapt to environmental changes and realize the mutual conversion between mechanical and electrical energy [17]. For example, the layered lead zirconate titanate (PZT) structure was prepared via freeze-casting, and the polydimethylsiloxane (PDMS) matrix was impregnated in arranged channels to form piezoelectric composites (Figure 2e) [18]. The structured PZT–PDMS composites exhibited a high effective longitudinal piezoelectric coefficient (d33*) of 750 pCN^−1^, which is higher than that of monolithic ceramic due to the combination of bending and flexural effects. The freeze-casting technology has the unique advantages of a low cost and simple fabrication, and it can produce three-dimensional piezoelectric structures with complex and arbitrary shapes.

### 2.6. Carbon Nanotubes

Compared with traditional conductive materials, carbon nanotubes have a large specific surface area, small size, high electron mobility, and high sensitivity to electrical interference of water or other gas molecules, and they have a very high position in the field of sensing. Most of the sensors made of carbon nanotubes and polymer composite materials have excellent mechanical properties, thermal stability, high conductivity, etc., so carbon nanotube–polymer composite materials are considered to be alternatives to traditional smart materials. Jin Wu et al. fabricated a humidity sensor using carbon nanocoils (cncs) on a flexible liquid crystal polymer (LCP) substrate. They have a fast response time (1.9 s), a short recovery time (1.5 s), and a wide relative humidity (RH) detection range (4–95%). These capabilities enable the sensors to accurately monitor a variety of important human activities, such as distinguishing between different human conditions by recognizing respiratory response patterns [19].

### 2.7. Other Materials

In addition to these representative materials, there are many materials that are used in the fabrication of multifunctional sensors. Multi-walled carbon nanotubes (MWCNTs) have unique geometric structures, excellent mechanical strength and electrical conductivity, and strong catalytic properties [20,21]. For example, Li et al. developed flexible and wear-resistant sensing electronics with multifunctional smart coatings [22]. The coating is a substrate with a superhydrophobic surface that responds to stretching, bending, and torsion. It also has the characteristics of high sensitivity, high resolution, a fast response time, stable response, and a wide sensing range. It can also be directly used in clothing as a wearable sensor to detect human motion in an all-around and real-time manner, showing extreme repulsion to water, acids, and bases, which helps the sensor to operate in wet and corrosive conditions. Xiong et al. developed a textile-based triboelectric nanogenerator driven by human movement through skin contact. It has a relatively high output (~250–880 V; ~0.48–1.1 μAcm^−2^). It can be touched by hand with a small force (~5 N) and low frequency (~4 Hz), which can power an LED and digital watch (Figure 2f) [23]. Using hydrophobic cellulose oleate nanoparticles coated with black phosphorus as a collaborative electron capture coating can make the textile nanogenerator work stably under extreme deformation, severe washing, and long-term environmental exposure conditions with good reliability and high tribological electricity. Liquid metal (LM) has been recognized as an ideal electrode material to manufacture electronic skin. Liquid metal refers to an amorphous metal, which can be seen as a mixture of positive ion fluids and free electron gases. Compared to most sensor materials, liquid metal materials are softer, can withstand greater strains, have no fatigue issues, and can self-heal when damaged. However, there are still certain difficulties, such as leakage risk, surface chemical instability, biocompatibility, and safety packaging issues [24]. Wonbeom et al. assembled a liquid metal particle network (LMPNet) by applying sound fields to solid insulated liquid metal particle composites as elastic conductors, which can tightly integrate many electronic components to create highly stretchable skin electronic components, and has great application potential in the field of soft electronics (Figure 2g) [25].

**Table 1:** Summary of multifunctional sensors: materials.

	Materials	Breathability	Temperature Range	Biocompatibility	Biodegradability	Ref.
1	Hydrogel material	good	0–15 °C	good	good	[6]
2	Graphene foam	good	−269∼1000 °C	average	good	[10]
3	Ferroelectric materials	poor	-	poor	average	[14]
4	Nano zinc oxide	poor	-	good	average	[15]
5	Ceramic-polymer composites	poor	1200∼1500 °C	poor	poor	[18]
6	Carbon nanotubes	average	-	average	good	[19]
7	Multi-walled carbon nanotubes	average	-	average	good	[20]
8	Liquid metal	poor	-	poor	poor	[24]

Overall, future research should expand to explore other types of sensors. Particularly, flexible optical sensors [26] and electrical impedance tomography (EIT) technology [27] rely on light matter interactions. EIT technology does not use ionizing radiation, it is non-invasive, and it has high temporal resolution. Optical sensors are very sensitive to the relative directions and motions of objects and play an increasingly important role in the development of medical diagnostic equipment. Optical sensors have excellent metrological characteristics, electromagnetic interference resistance, electrical safety, simple miniaturization, the ability to capture nano volumes, and non-invasive inspection. In addition, they are inexpensive, waterproof, corrosion-resistant, and so on.

Sensing technology is an application technology that utilizes various functional materials to achieve detection purposes. Various functional materials are the material foundation for the development of sensing technology, and the research and development of new sensing technologies cannot be separated from the application of new materials.

### 2.8. Biocompatibility

Biocompatibility is the ability of living tissues to react to non-active materials. After biomaterials are implanted into the human body, they have an impact and effect on the specific biological tissue environment, and the biological tissue will also have an impact and effect on the biological material, and the cyclic effect of the two continues until equilibrium is reached or the implant is removed. Biocompatibility is an important topic in the research of biological interface materials. The location and duration of contact between the material and the tissue need to be considered in the experimental process, because the biological effects of the material/device on the tissue vary greatly with these two factors [28].

Biocompatibility focuses on the immune response, which varies from person to person and needs to be carefully considered, and it is not only related to the safety and acceptance of the user, but also to the performance of the sensor. Second, for immune responses with no obvious harm, such as the formation of fibrosis, no serious inflammation occurs, but the performance of the sensor is greatly reduced. In this case, the immune response must not only be suppressed, but also eliminated. 

At present, there are many differences in the biocompatibility of nanomaterials such as graphene and carbon nanotubes. This problem is partly due to the non-standardized testing in different studies, and partly due to the huge changes in the properties of nanomaterials due to the variety of sizes and geometries in the synthesis process [29]. It will take a long time to standardize the biocompatibility of nanomaterials.

## 3. Structure and Principle 

The structure can influence the function, scope of application, and accuracy of a sensor. When it adopts different designs, even with the same material, the final sensing performance of the sensor will be improved.

### 3.1. Matrix Structure

**Figure 3:**
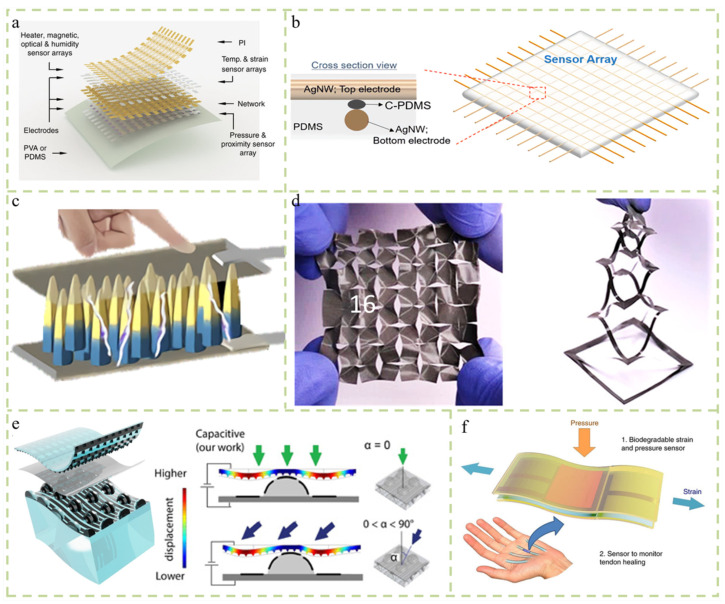
Sensors with different design structures and principles. (**a**) A highly stretchable and conformal matrix network inspired by skin enables simultaneous multi-stimulus sensing [30]. (**b**) A highly stretchable cross-reaction sensor matrix that can detect, classify, and distinguish various mixed tactile and thermal stimuli using machine learning methods [31]. (**c**) Synthesized ZnO nanorod arrays and constructed strain sensors with high sensitivity, flexibility, wearability, and chemical stability [32]. (**d**) Layered bionic electronic skin composed of capacitor array that can measure and distinguish normal force and tangential force in real time [34]. (**e**) An electronic skin designed with liquid metal and paper-cutting art can simultaneously realize the self-support, conductor exposure, stretching, ultra-thin, and recyclable characteristics [36]. (**f**) Implantable input pressure and strain sensors made entirely of biodegradable materials [37].

A mechanosensory electronic device (or electronic skin, e-skin) consists of a network of mechanically flexible and stretchable sensors that mimic the somatosensory system of the human skin, realizing the functionality of detecting pressure and temperature. For example, Hua et al. designed a matrix array of nodes connected to a zigzag line, which is highly stretchable and comfortable to wear with the sensing function of temperature, plane strain, and relative humidity (RH) (Figure 3a) [30]. The durability test showed that the sensors had very good stability and durability. Previously, field effect transistors composed of piezoelectric/thermoelectric gate dielectrics and piezoelectric organic semiconductor channels were used to achieve simultaneous responses to both pressure (or strain) and temperature stimuli. To distinguish mixed signals composed of known and unknown stimuli, a highly stretchable cross-reactive sensor was designed in a 10 × 10 matrix consisting of cross-arranged highly stretchable electrodes and multiple electrodes sandwiched between the electrodes [31]. It consists of a mode hybrid sensing element that can respond quickly to various stimuli such as strain, pressure, bending, and temperature with high sensitivity and crossover operation modes (Figure 3b). The discrimination of different stimuli was achieved by implementing multimodal perception using a bag-of-words (BoW)-model-based machine learning algorithm, which distinguished each stimulus by recognizing different 2D image patterns generated by mixed tactile and thermal stimuli.

### 3.2. Three-Dimensional Structure

Controlling the precise structure of synthesized 1D to 3D nanomaterials is a key factor in improving the sensitivity of the sensor. Yu et al. reported a natural biomimetic approach to synthesize zinc oxide nanorod (ZnONR) arrays in situ on graphene-treated cotton and paper substrates, enabling the creation of the 3D structure of nanorods with piezoelectric potential under a bent force (Figure 3c) [32]. Yang et al. developed a new type of porous pyramidal dielectric layer (PPDL), which is insensitive to strain and temperature changes and can detect pulses and weak airflow [33]. Xing et al. designed an electronic skin using liquid metal and paper-cutting art, which has super tensile properties, is ultra-thin, and has recyclable materials. It provides potential in areas such as medical monitoring and intelligent control, intelligent robots, and the use of personal electronic products on the skin (Figure 3d) [34].

### 3.3. Hierarchical Structure

Simulating the interlocking dermal–epidermal interface in human skin is an effective method to develop electronic skin [35]. Boutry et al. developed a biomimetic soft electronic skin that can measure and distinguish between normal and tangential forces in real time (Figure 3e) [36]. The top layer of the electronic skin is a molded square pyramid network arranged in a nature-inspired phyllotaxy spiral. When external pressure is applied, elastic deformation is produced, and energy is reversibly stored and released, thereby increasing sensitivity. The bottom layer of the electronic skin consists of a two-dimensional (2D) array of molded hills that mimic the spinous layers of human skin, and they are critical for measuring and discerning the direction of applied forces. The hierarchical structure can improve the good sensitivity, cyclic stability, small hysteresis effect, and fast response time of the electronic skin. Boutry et al. developed a flexible strain sensor with two stacked structures, allowing for the independent identification of strain and pressure [37]. The design with the requirements to achieve high sensitivity and fast response can adequately adopt organic materials in the application of human tissue rehabilitation monitoring (Figure 3f). Table 2 summarizes some other types of sensors with different structures. Through a comparison, it can be seen that even the same material may have different detection ranges, sensitivities, and other property differences in application due to differences in the design structure. At the same time, it also reflects the importance of structure in multifunctional sensor design.

## 4. Different Stimulus Detection

**Figure 4:**
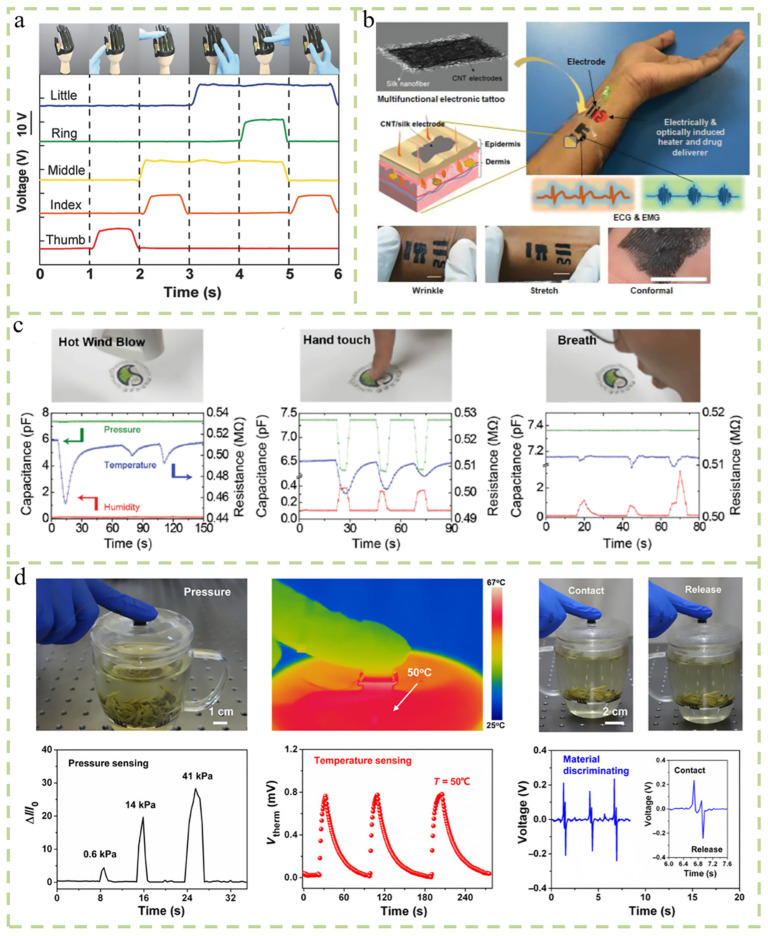
Sensor detection for different stimuli. (**a**) The characteristics of STPS are sensed by integrating on the robot hand [48]. (**b**) Single component for multi-stimulus detection achieved by dispersing highly conductive carbon nanotubes onto a nanofiber network [49]. (**c**) The simultaneous sensing performance of the sensor under different stimuli of hot air and hand touch [50]. (**d**) After physical contact with different planar materials, an output voltage signal can be generated to infer material characteristics [52].

Developing sensors that are capable of simultaneously measuring multiple mechanical and chemical stimuli is crucial for human–computer interaction and health monitoring. Bu et al. designed a tactile sensor to realize multi-dimensional tactile and gesture perception of robot hands. It measures strain and pressure via the light response and friction voltage generation [48]. When the strain was applied, designed microcracks appeared in the metal film on top of the induced emission (AIE) compound, and the photocurrent generated by the AIE increased. The sensor can also be used as a TENG-based pressure sensor to guarantee an accurate response regardless of changes in the applied strain (Figure 4a). Gogurla et al. utilized natural silk proteins and carbon nanotubes (CNTs) to achieve multifunctional applications of electronic tattoo systems. The electronic tattoo was constructed by dispersing highly conductive carbon nanotubes onto a porous and biocompatible silk nanofiber network, achieving a single component consisting of an electric and optical active heater, temperature sensor, drug delivery stimulator, and real-time electrophysiological signal detector (Figure 4b) [49]. Through a simple laminating process, Ho et al. integrated three different functional sensors, namely humidity, temperature, and pressure sensors, into a layer-by-layer geometric structure (Figure 4c) [50]. The design of multifunctional sensor systems has been studied in two ways: using a single sensor module that responds to multiple stimuli and integrating multiple response modules together [51]. Each sensor in the matrix exhibits a single sensing performance. It is only sensitive to a specific stimulus and does not respond to other stimuli. When exposed to external stimuli, the three sensors in the matrix simultaneously detect external stimuli, transmit independent electrical signals, and collect 2D color images. Synchronous multifunctional sensing is a multifunctional sensor integration using a simple lamination method (Figure 4d) [52]. 

## 5. Self-Powered Sensors

With the rapid development of sensor and electronic component integration, the design and development of a stable power supply and an efficient system has become mainstream. Self-actuated sensors have environmental protection capabilities, portability, low costs, and long service lives. 

Friction nanogenerator (TENG) is a new energy conversion technology that utilizes the electrostatic energy generated by friction to convert it into usable electrical energy. The research and application of TENG friction nanogenerators are of great significance, as they can provide green and sustainable power supply for wearable devices, mobile communication, and other fields. Similarly, friction nanogenerators also face some difficulties in practical applications. Firstly, their energy conversion efficiency and output voltage need to be further improved to meet the needs of high-energy-consuming devices. Secondly, the stability and reliability of friction nanogenerators also need to be strengthened to ensure long-term continuous operation. In addition, for the application needs of different work scenarios, it is also necessary to design and optimize friction nanogenerators of different structural types.

A piezoelectric nanogenerator (PENG) is an emerging energy technology based on the Maxwell displacement current that is used to collect mechanical energy and directly perceive external mechanical stimuli, providing feasibility for various self-powered sensing systems. However, the inherent weak piezoelectric effect of polymers leads to a low piezoelectric output and an unstable signal of the prepared PENG, which is limited by the size of the equipment, and it is difficult to achieve the required piezoelectric performance. The large-scale preparation of polymer PENGs with high sensitivity and a stable piezoelectric output remains a great challenge.

**Figure 5:**
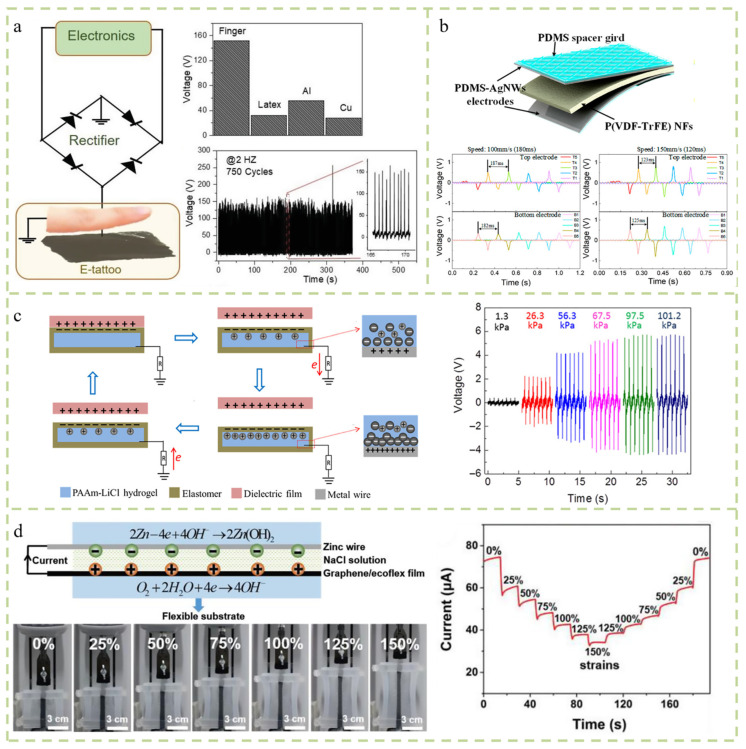
Self-powered sensors. (**a**) A self-powered electronic tattoo sticker composed of carbon nanotubes (CNTs) and nanowire fibers (SNFs) [53]. (**b**) A new active multifunctional electronic skin capable of independently detecting contact trajectory, acceleration, velocity, and pressure [54]. (**c**) Biomechanical energy collection and tactile sensing are realized by using mixed elastomer and ionized hydrogel as charged layer and electrode, respectively [55]. (**d**) Graphene/ecoflex film and zinc wire integrated into a flexible substrate. A built sensor that not only has good tensile performance, but can also obtain stable current and voltage signals [57].

They can convert various energies in the environment into electrical energy and replace batteries to a certain extent. In addition to the high requirements for energy, they are also very demanding in terms of portability and wearability. There are many energy sources in the environment. Gogurla et al. proposed a self-powered electronic tattoo sticker composed of carbon nanotubes (CNTs) and nanowire fibers (SNFs). This electronic tattoo can be unknowingly tattooed on the skin and then removed with water. When the device comes into contact with exposed skin, it generates frictional electrical signals. The energy that is generated can activate small electronic devices and monitor joint movements throughout the body (Figure 5a) [53]. Guo et al. reported a self-powered electronic skin consisting of triboelectric nanogenerators (TENGs) and piezoelectric nanogenerators (PENGs). When an object moves on the surface of a device, it can be used without external connections based on the piezoelectric effect caused by friction and the origin of friction [54]. The motion information of objects can be detected under the condition of a power supply (Figure 5b). Pu et al. reported a soft skin-like triboelectric nanogenerator that used elastomers and ionic hydrogels as charged layers and electrodes to harvest biomechanical energy and enable tactile sensing [55]. The material is highly stretchable and transparent. The energy harvested through human movement can power small electronics that replace power sources (Figure 5c). Piezoelectric generation is one of the widely used mechanisms for self-powered sensors. Zhang et al. achieved the simultaneous monitoring of the temperature and pressure by converting external stimuli into separate electrical signals using independent thermoelectric and piezoresistive effects in a single MFSOTE device [56]. These devices can be self-powered by natural temperature gradients with a high temperature resolution and high pressure sensitivity. It has a broad application prospect in artificial intelligence and the healthcare system. Yang et al. designed a new type of strain sensor that integrates graphene/ecoflex film and zinc wire into a flexible base. The constructed strain sensor not only has good tensile performance, but can also generate oxidation–reduction-induced current signals, which can obtain stable current and voltage signals (Figure 5d) [57].

## 6. Wireless Communication

**Figure 6:**
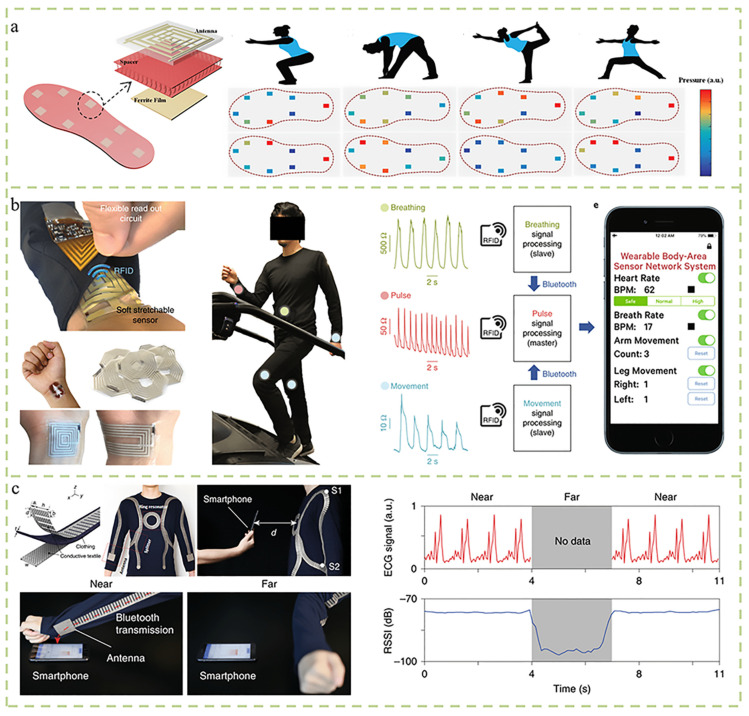
Wireless communication for sensor. (**a**) A textile-based wireless pressure sensor array based on a fabric spacer sandwiched between a passive antenna and a ferrite film [60]. (**b**) A wireless body area sensor network based on stretchable passive tags consisting of chips and battery-free stretchable skin sensor tag [61]. (**c**) Energy-efficient and safe wireless body sensors with radio surface plasmon interconnections propagating on metamaterial textile network [62].

The data obtained through measurement should be accurate and convenient to explain the problem in practice. Wireless signals are widely used in wearable devices due to their convenient communication and freedom of movement. Common wireless communication technologies include electromagnetic coupling technology [58], Bluetooth communication [59], and passive radio frequency identification (RFID) technology. Electromagnetic coupling occurs between the internal LC or RLC resonator (consisting of inductors, capacitors, and resistors in series) and the external resonator connected to the readout system. The applied excitation changes the effective inductance or capacitance of the internal resonator. The shift in the resonant frequency of the internal circuit caused by the external stimulus can be detected by reading the shift or magnitude of the reflection coefficient of the circuit analyzer. This approach simplifies the connection of appliances and reduces the volume. Electromagnetic coupling has been widely used in a variety of wireless sensing applications. Nie et al. designed a fabric gasket stacked between the LC antenna and the ferrite film, applying pressure to deform the fabric gasket and reduce the distance between the antenna and the ferrite film, leading to the resonant frequency shift of the LC antenna. Sensors can be attached to a flexible wristband, shoe insole, or belt to showcase a variety of remote applications (Figure 6a) [60]. Passive radio frequency identification technology is a radio wave non-contact identification technology, which can quickly collect and store information and conduct non-contact data communication between the reader and the tag. For example, it is based on a human body network system of stretchable sensors attached to multiple skin locations to collect human physiological and movement signals that are wirelessly operated via silicon readout circuits attached to textiles (Figure 6b) [61]. The physically separated stretchable sensor and silicon readout circuit communicate via passive radio frequency identification (RFID) technology. Strain-induced changes in the sensor antenna inductance and resistance are addressed by employing unconventional detuned RFID tag designs. Connected to a mobile phone, it can be monitored in real time, thereby realizing remote detection. Tian et al. found that clothing constructed from conductive textiles can support surface plasmon patterns at communication frequencies, thus providing a platform for radio waves to propagate around the body (Figure 6c) [62]. In this way, energy-efficient and secure wireless human sensor networks via radio surface plasmon interconnects propagated over metamaterial textiles have been developed. The contactless proximity to the body enables the interconnection of devices, and the physical positioning of wireless signals on the body enables efficient, undisturbed, and secure personal sensor networks.

## 7. Application

**Figure 7:**
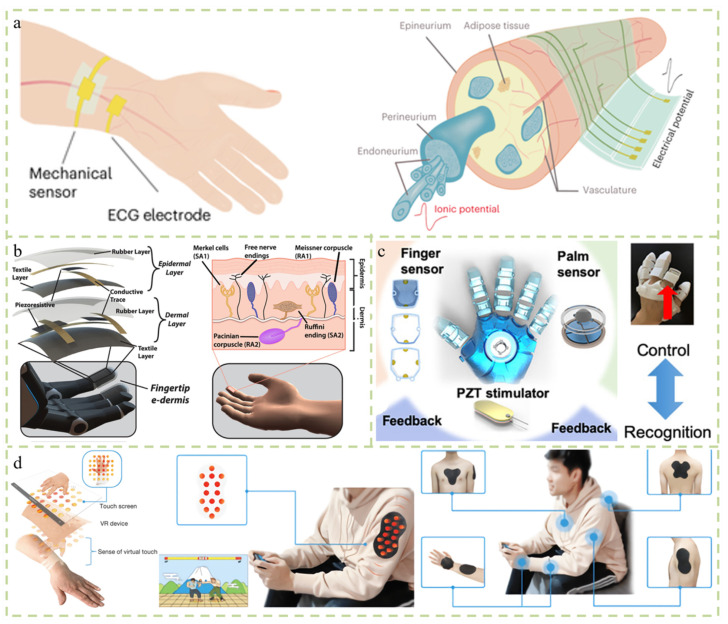
Application. (**a**) Polydimethylsiloxane (PDMS) film and gold conductor are used to manufacture breathable and waterproof skin electrodes, which can continuously record electrocardiogram signals in daily life and exercise [67]. (**b**) A neuromorphic multi-layer electronic dermis (e-dermis), with properties based on the behaviors of mechanoreceptors and nociceptors to provide neuromorphic haptic information to amputees [68]. (**c**) Haptic feedback smart gloves used to provide haptic feedback in interactive human–machine interfaces and VR/AR applications [70]. (**d**) Skin-integrated wireless haptic interfaces for virtual and augmented reality, such as epidermal wireless haptic sensing for prosthetics and VR/AR applications [71].

The design and development of multifunctional sensors expanded and enriched people’s daily lives and work. Ergonomics and tactile performance are essential considerations to achieve wearable tactile devices that are capable of effective data communication [63]. In terms of wearing, tactile devices should meet the compliance and comfort requirements of daily clothing. This study proposes a tactile device made entirely of textiles—a squeeze band [64]. This fabric-based device is actuated via pressurized air and achieves a pressure response by changing the geometric shape of the inflatable area inside the device. Fabrics woven from specially prepared coaxial yarns are also used as sensing surfaces. The capacitive smart sensor fabric also achieves the goal of comfortable wearing and superior performance [65]. Robots equipped with tactile sensors and feedback systems can perform more sophisticated functions than robots that rely solely on visual perception. It has an excellent performance in detecting hazardous materials and grasping dangerous and fragile objects. Lim et al. reported a human–computer interface consisting of wearable mechanical sensors and actuators [66]. The epidermal sensor is composed of a polymer and nano-material wires. After wearing the system, it can detect signals, such as the bending and relaxation of the human arm, and the signals will be transmitted to the control terminal to control the robot. Similarly, when the robotic arm detects an object, it will provide feedback to alert the user. Zhi et al. utilized polydimethylsiloxane (PDMS) gold conductors with a thickness of approximately 1.3 μm to create breathable and waterproof skin electrodes that can continuously record ECG signals in daily life, as well as during intense sports such as running and swimming. A stretchable sensor with a thickness of 3 microns can be made, which can not only detect small mechanical forces, but can also be used to make implanted sensing and stimulation electrodes (Figure 7a) [67]. Osborn et al. designed a multi-layer electronic dermis based on mechanoreceptors and nociceptors, which enables the prosthetics and their users to perceive a continuous spectrum from harmless to harmful through the neuromorphic interface, providing amputees with neuromorphic tactile information [68]. Amputees can use touch and pain perception to distinguish objects that are sharp or not (Figure 7b). Simply receiving data feedback is not enough. Virtual reality (VR) and augmented reality (AR) systems have recently received widespread attention due to their improved accessibility, functionality, and affordability [69]. By connecting machines and humans through VR or AR technology, human–machine interaction can be achieved and gradually attract more people’s attention. Zhu et al. designed and developed a tactile feedback smart glove that is suitable for VR or AR (Figure 7c) [70]. The multi-directional bending and sliding are detected by triboelectric signals generated through the bending of the wearer’s hand and transmitted to the virtual space. At the same time, the piezoelectric chip is used for tactile mechanical stimulation to enhance the human–computer interaction experience. The production cost is low, the human–computer interaction sensitivity is high, and the application prospects in entertainment, sports, medical, and other aspects are wide. Yu et al. developed a wearable haptic display with wireless control and powered vibration actuators. It has applications in the tactile feedback of prosthetic limbs and the control of game characters in real-time reproduction (Figure 7d) [71].

## 8. Conclusions and Outlook

Multifunctional sensors can respond to various stimuli, which play important roles in many applications, including electronic skin and medical diagnosis. In these applications, there is an increasing demand for high-performance multifunctional sensors. Researchers have reported a number of guiding strategies to address this requirement. In this review, we highlighted the recent attempts to fabricate higher-performing multifunctional sensors. The attempts include the use of functional materials, advanced structures, and intelligent systems, all of which present distinct capabilities of improving the performance of the sensors. These include linearity, sensitivity, and detecting variety. 

While several methods that can achieve outstanding multifunction sensing have been presented in this paper, there are still some challenges in industrial applications. These challenges include the following: (1) the development of nanomaterials with good mechanical compliance and physicochemical properties; (2) device designs that establish a balance between detection sensitivity and sensing functions; (3) data management methods that are compatible with artificial intelligence systems; and (4) a high-level large-area integration of sensor arrays. 

In summary, the above challenges in multifunctional flexible sensors need collective efforts from science researchers to industrial engineers. Coupling experimental validation with fabrication innovations will help to solve these problems. We hope that this review will provide an extensive understanding of the developments, thus helping in the design of advanced sensors in the future. 

## Figures and Tables

**Table 2:** Summary of multifunctional sensors: types of devices and properties.

	Types of Devices	Sensitivity	Examination Area	Limit of Detection	Response Time	Ref.
1	Spongy PDMS film dielectric layer	0.63 kPa^−1^	<1 kPa	2.42 Pa	40 ms	[38]
2	Microstructured drain/source electrodes	514 kPa^−1^	30∼200 Pa	10 Pa	1.8 ms	[39]
3	Tilted micropillar array	0.42 kPa^−1^	<1.5 kPa	1 Pa	<10 ms	[40]
4	Pyramid porous structure	44.5 kPa^−1^	100 Pa	0.14 Pa	100 ms	[41]
5	Hierarchical structure	26.13 kPa^−1^	0.2∼982 kPa	982 kPa	83 ms	[42]
6	Interlocking ZnO microparticles	75∼121 kPa^−1^	0–200 Pa	0.015 Pa	0.015 ms	[43]
7	Suspended gate transistor	192 kPa^−1^	100–5000 Pa	<0.5 Pa	10 ms	[44]
8	Hollow sphere microstructure	133.1 kPa^−1^	——	0.8 Pa	47 ms	[45]
9	Graphene array	5.53 kPa^−1^	0∼100 Pa	1.5 Pa	0.2 ms	[46]
10	PDMS mimosa micro graphics	50.17 kPa^−1^	0∼70 Pa	10.4 Pa	<20 ms	[47]
11	ZnO nano sea urchin structure	121 kPa^−1^	0∼200 Pa	——	7 ms	[43]
